# Supplementary material for: Short-term responses of temperate and subarctic marine diatoms to Irgarol 1051 and UV radiation: Insights into temperature interactions
Source: PLoS One. 2024 Feb 7;19(2):e0295686. doi: 10.1371/journal.pone.0295686 (PMC10849241; doi:10.1371/journal.pone.0295686)
Supplement: Attachment — Submitted filename: Response.docx [file pone.0295686.s003.docx]

Reviewer #1: Authors: there are indeed some publications available for Thalassiosira weissflogii and Skeletonema costatum, while there are relatively fewer studies on Entomoneis sp. It is important to note that comparing the sensitivities of these species can be challenging due to variations in the experimental conditions used in different studies.

My answer: Yes, it's difficult to compare work performed under different conditions, but if there are a lot of studies, similar behaviour can sometimes emerge between them. I think you can at least refer to the publications you're talking about for these species (if only to say that there are a few studies for the first two and fewer data for the last one).

Response: We have added a sentence to clarify this point at line 93-95, and related references have been added.

Reviewer #2: I acknowledge the changes, comments and precisions that were included in the revised manuscript, in my opinion making it suitable for acceptance.

I have just one suggestion, to keep an important feature of the presented study - in my opinion - change the title for: "Short-term responses of temperate and subarctic marine diatoms to Irgarol 1051 and UV radiation: Insights into temperature interactions"

Response: Thank you for the suggestion. We agree with the reviewer and have made the necessary changes. The title has been updated accordingly, and the term "cold-water" has been replaced with "subarctic" throughout the entire text.
